# Site-Specific Photochemical Reaction for Improved C=C Location Analysis of Unsaturated Lipids by Ultraviolet Photodissociation

**DOI:** 10.34133/2022/9783602

**Published:** 2022-02-12

**Authors:** Hai-Fang Li, Jing Zhao, Wenbo Cao, Wenpeng Zhang, Yu Xia, Zheng Ouyang

**Affiliations:** ^1^State Key Laboratory of Precision Measurement Technology and Instruments, Department of Precision Instrument, Tsinghua University, Beijing 100084, China; ^2^MOE Key Laboratory of Bioorganic Phosphorus Chemistry & Chemical Biology, Department of Chemistry, Tsinghua University, Beijing 100084, China

## Abstract

Unraveling the complexity of the lipidome requires the development of novel approaches to facilitate structural identification and characterization of lipid species with isomer-level discrimination. Ultraviolet photodissociation tandem mass spectrometry (UVPD MS/MS) is a promising tool for structure determination of lipids. The sensitivity of UVPD for lipid analysis however is limited mainly due to weak absorption of UV photons by a C=C. Herein, a C=C site-specific derivatization, the Paternò-Büchi (PB) reaction, was used to incorporate a chromophore to the C=C moiety in fatty acyls, leading to significantly improved UVPD efficiency and sensitivity for pinpointing C=C locations. The wavelength-dependent photodissociation of the PB products demonstrated 4-CF_3_-benzophenone as the best reagent for UVPD in terms of the efficiency of generating C=C diagnostic fragments and simplicity for C=C location assignments. We demonstrated the effectiveness of this approach for the shotgun profiling of C=C location isomers in different lipid classes from complex lipid extracts, highlighting its potential to advancing the identification of the C=C bond locations in unsaturated lipids.

## 1. Introduction

Lipids are an essential class of biological molecules that exhibit numerous vital functions in living organisms, including membrane matrices, energy storages, cellular signalling [1, 2]. Unsaturated lipids, which are characterized by the presence of one or multiple carbon–carbon double bonds (C=Cs) in fatty acyl chains, constitute a large number of individual lipid species. The various degrees of unsaturation and diversiform C=C locations in fatty acyls contribute to the complexity of unsaturated lipids [3, 4]. Dysregulation of the unsaturated lipid profile is associated with several diseases, such as type-2 diabetes, cancer, and cardiovascular disease [5–8]. Thus, it is important to characterize the lipid C=C locations and distinguish isomers in order to further understand their biological roles.

Tandem mass spectrometry (MS/MS) has become the enabling tool for lipid profiling because of its high sensitivity and specificity in lipid identification and quantitation [9]. Collision-induced dissociation (CID) is the most frequently used MS/MS technique [10], and it is capable of providing lipid head group and fatty acyl chain composition for complex lipids. However, the information regarding the C=C locations within the fatty acyl chains was hardly provided through CID MS/MS alone [11, 12]. Thus, site-specific derivatization on C=C bonds, such as epoxidation [13–16], singlet oxygen ene reaction [17], and the Paternò-Büchi (PB) reaction [18, 19], have been developed to enable double bond localization in conjunction with CID. Ultraviolet photodissociation (UVPD) as an alternative ion activation method has been utilized independently for providing detailed structural characterization of intact lipids [20–22]. UVPD MS/MS at 193 nm has previously been implemented in shotgun and HPLC workflows capable of assigning *sn*-positions of fatty acyls and resolving C=C positional isomers of glycerophospholipids (GPLs) [23, 24] and sphingolipids [25]. The distinct diagnostic ions, resulting from the cleavage of C−C adjacent to a C=C in acyl chains from UVPD, serve as a signature for tracing the original C=C bond positions. 157 nm UVPD has also been used to reveal structural differences in isomeric lipids of leukotriene [26]. The 213 nm UVPD MS/MS, now commercially incorporated on a Thermo Scientific Orbitrap Fusion Lumos MS, allows locating cyclopropane rings [27], ester positions, and C=C locations in fatty acid esters of hydroxy fatty acid (FAHFA) from UVPD of the resulting fatty acid ions from the dehydration of FAHFA with HCD [28], albeit with reduced fragmentation efficiency. Owing to the absence of a strong chromophore in the lipids, the above UVPD MS/MS suffers from low intensities of diagnostic fragments [24]. It is desirable to adopt an efficient approach to achieve a red shift of wavelength above 200 nm for UVPD and the concomitant improved intensities of fragment characteristic to the key structural features of the lipids. An alternative strategy deploys 266 nm UVPD of C−I bond, a highly efficient and selective chromophore, in derivatized lipid to initiate radical-directed dissociation (RDD) [29, 30]. RDD has been shown to be capable of characterizing C=C bond [31] and methyl branching positions [32], as well as the hydroxyl groups [33].

Recently, the PB reaction has been adopted as a C=C-specific derivatization method for analysis of unsaturated lipids [34, 35]. Besides acetone, aryl ketones [36–40] and aldehyde [41], which have much higher molar absorptivity than acetone, have been applied in the PB reaction of unsaturated lipids. Most of the developed PB-MS/MS workflows employed CID technique to generate C=C diagnostic fragments. Wäldchen et al. showed that it was possible to couple the acetophenone-PB reaction and 266 nm UVPD for C=C location analysis of phosphatidylcholines (PCs) [36]. Several key experimental factors critical to assessing the analytical potential of the PB-UPVD approach however remain unclear. These include the following: (1) can one rationally design a better PB reagent than acetophenone? (2) Is the efficiency for generating C=C diagnostic ion wavelength-dependent? (3) Is PB-UVPD an approach that can be applied to different classes of lipids?

**Figure 1:**
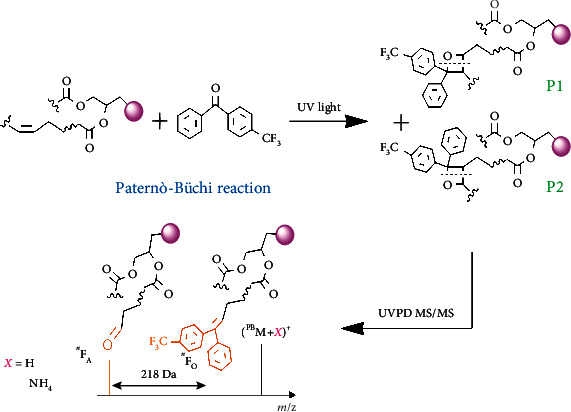
Schematic presentation of pinpointing C=C locations in unsaturated GPLs by coupling the in-solution PB reaction with UVPD MS/MS.

In this study, we utilized wavelength-tunable UVPD (Figure S1) with a wide range of PB reagents. 4-CF_3_-benzophenone (4-CF_3_-Bzp) was identified as the best reagent in terms of generating C=C diagnostic fragments in high efficiency via UVPD MS/MS (Figure 1). Coupling the in-solution PB reaction with UVPD was found powerful for structural informative lipidomics analysis, which enabled C=C isomer identifications for a variety of GPLs from lipid extracts of bovine liver and *Escherichia coli* (*E. coli*) using a shotgun analysis approach.

## 2. Results and Discussion

### 2.1. Coupling the PB Reaction with UVPD MS/MS

**Figure 2:**
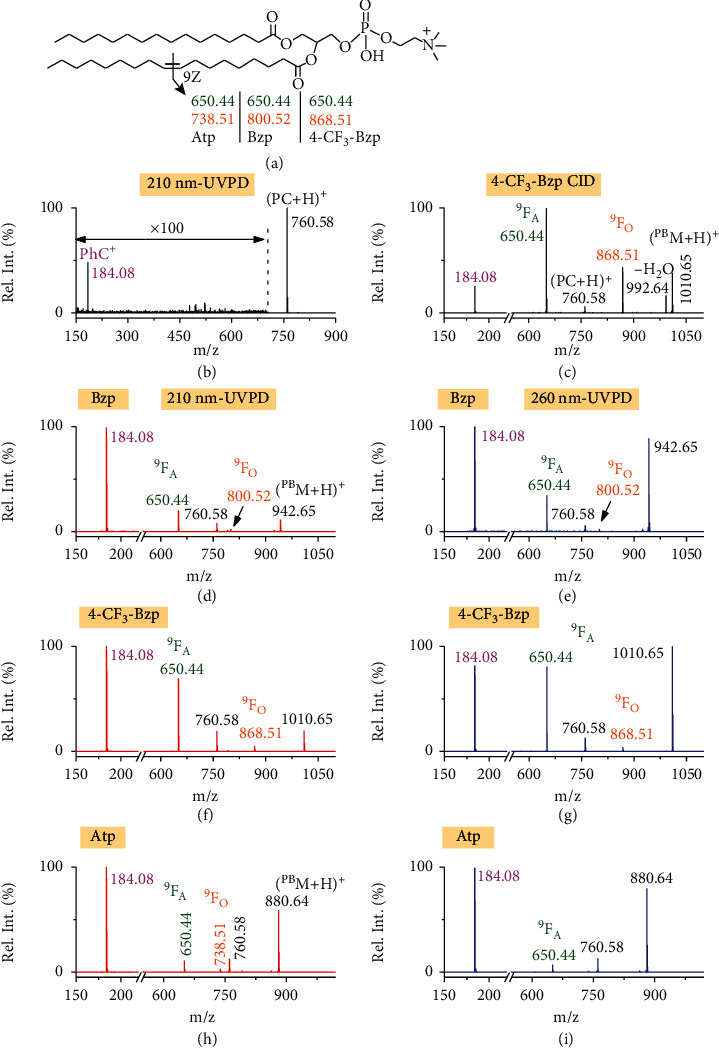
(a) The structure of PC 16 : 0/18 : 1 (9Z) and the C=C diagnostic fragments generated from Atp, Bzp, or 4-CF_3_-Bzp as the PB reagents; (b) 210 nm UVPD MS/MS spectrum of PC 16 : 0/18 : 1 (9Z) after 10 pulses; (c) CID MS/MS spectrum of PB product ions of PC 16 : 0/18 : 1 (9Z) derivatized by 4-CF_3_-Bzp with collision energy applied at 25 eV; UVPD MS/MS spectra of PB products of PC 16 : 0/18 : 1 (9Z) with (d, e) Bzp, (f, g) 4-CF_3_ Bzp, or (h, i) Atp at 210 nm and 260 nm after 2 pulses. The laser energies were 0.5–1.0 mJ per pulse. C=C diagnostic ions denoted as ^9^F_A_ and ^9^F_O_ were labeled as olive and orange, respectively. Pulse-dependent efficiencies for photodissociation of Bzp or 4-CF_3_-Bzp modified PC 16 : 0/18 : 1 (9Z) lipid are given in Figure S3.

Recently, Zhao et al. [42] found that the substitution of electron-withdrawing groups (e.g., −F and −CF_3_) on the benzene ring improved the overall conversion efficiency of acetophenone as the PB reagent. Therefore, benzophenone (Bzp), acetophenone (Atp), and the corresponding −CF_3_-monosubstituted derivatives were chosen as the PB reagents to test their coupling with UVPD. The glycerophospholipid 1-palmitoyl-2-oleoyl-*sn*-glycero-3-phosphocholine (PC 16 : 0/18 : 1 (9Z), the structure shown in Figure 2(a)) was used as a model compound for evaluating UVPD MS/MS of the PB derivatized lipids. We first tested 210 nm UVPD of the intact PC ions ([PC+H]^+^), the shortest wavelength of the tunable laser. The UVPD fragmentation efficiency was low, and no characteristic fragments relating to C=C locations were experimentally observed even with 10-pulse irradiation (trapping time: 500 ms, Figure 2(b)). Similar phenomenon was observed with 260 nm UVPD of PC 16 : 0/18 : 1 (9Z) (Figure S2). These data suggest that the efficiency of 210 nm and 260 nm UVPD is too low to deliver sufficient fragmentation around C=C. As a contrast, 210 nm UVPD of the PB products ([^PB^*M*+H]^+^, *m*/*z* 942.65) using Bzp as the reagent produced 4 fragment peaks (Figure 2(d)) with 2-pulse irradiation (trapping time: 100 ms). It is noteworthy that 2-pulse irradiation was found to be optimal for UVPD of PB products with Bzp reagent from the results of the pulse-dependent measurements (Figure S3). In this spectrum, protonated intact lipid [PC+H]^+^ and phosphocholine ions (PhC^+^) were observed at *m*/*z* 760.58 and *m*/*z* 184.08, respectively. Most importantly, two C=C diagnostic ions with a mass difference of 150 Da were observed at *m*/*z* 650.44 (^9^F_A_) and *m*/*z* 800.52 (^9^F_O_), which were formed from breaking the two oxetane ring region isomers (P1 and P2, Figure 1). When the wavelength used for UVPD MS/MS analysis was moved from 210 nm to 260 nm, the above assigned fragments were also observed, but at an increased ratio of *I*(^9^F_A_) over *I*(^9^F_O_) with respect to that from 210 nm UVPD (Figure 2(e), 6.5 vs. 11.1). These results demonstrate that incorporating chromophore to lipid via the PB reaction can significantly facilitate UVPD efficiency for lipid analysis by employing wavelength longer than 200 nm.

To obtain a potential PB reagent capable of generating increased abundances of diagnostic fragments for C=C locations with UVPD MS/MS, we also studied the UVPD MS/MS of the model PC compound derivatized by Bzp monosubstituted with −CF_3_ in *ortho* (2-), *meta* (3-), and *para* (4-) position. The PB-UVPD from the studied three reagents all generated PhC^+^, [PC+H]^+^, and C=C diagnostic ions (Figure S4 and Figures 2(f) and 2(g)). However, the relative ion abundances of C=C diagnostic ions were increased, especially for the ^9^F_A_ ions (*m*/*z* 650.44) in the order of *ortho* (2-), *meta* (3-), and *para* (4-) substitution. Similar to that observed from Bzp, the relative ion abundance of ^9^F_A_ was more abundant than that of ^9^F_O_. *I*(^9^F_A_)/*I*(^9^F_O_) was 11.2 from 210 nm UVPD while it went up to 22.0 under 260 nm when 4-CF_3_-Bzp was used as the PB reagent. This could be attributed to the molar 260 nm UV absorptivity of the P1 products (Figure 1) is higher than that of P2, and therefore, the intensity of ^9^F_A_ ions is higher than that of the ^9^F_O_ ions. In contrast, at 210 nm, the UV absorptivity of P2 is higher than that of P1. Considering the population of IS1 isomer is more than that of IS2 in the ion trap for UVPD (the energy of IS1 structure is lower than that of IS2), the relative intensities of ^9^F_A_ ions were still characterized to be higher than that of ^9^F_O_ ions. However, *I*(^9^F_A_)/*I*(^9^F_O_) went down from 22.0 under 260 nm to 11.2 under 210 nm (see the detailed discussion about *I*(^9^F_A_)/*I*(^9^F_O_) at 210 nm and 260 nm UVPD in Figure S5). Overall, we found 4-CF_3_-Bzp was a desirable reagent for enhancing the formation of the C=C diagnostic ions using a different UV wavelength. As a comparison, when CID MS/MS was conducted for the PB products of PC 16 : 0/18 : 1 (9Z), an additional peak (*m*/*z* 992.64) due to loss of H_2_O from PB products was observed (Figure 2(c)) [7]. These results suggested that the C=C site-specific chromophore-tagging derivatization allows a certain degree of selectivity of fragmentation in combination with UVPD MS/MS.

Atp was also tested, and the UVPD MS/MS of the resulting PB products of PC 16 : 0/18 : 1 (9Z) showed a similar fragmentation pattern to Bzp, albeit affording even lower relative ion abundance of C=C diagnostic ions (Figures 2(h) and 2(i)). Substitution of −CF_3_ on Atp did not afford an obvious increase for the formation of C=C diagnostic ions in UVPD (Figure S6).

**Figure 3:**
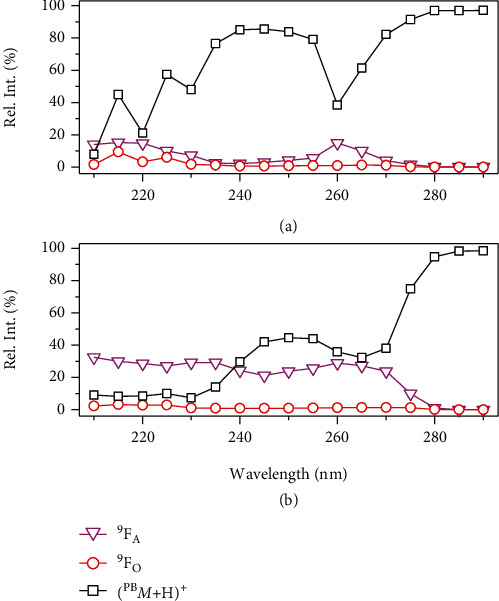
Wavelength-dependent photodissociation profiles of PB products of PC 16 : 0/18 : 1 (9Z) with (a) Bzp or (b) 4-CF_3_-Bzp after 2 pulses. The product ions ([^PB^*M*+H]^+^) and C=C diagnostic ions (^9^F_A_ and ^9^F_O_) are highlighted. The wavelength-dependent relative ion intensities of other fragment ions [PC+H]^+^ and PhC^+^ are given in Figure S7.

The wavelength-dependent photodissociation [43] was employed to determine an optimal wavelength to facilitate C=C locations. The relative intensities of parent and photofragment ions were collected by varying the wavelength from 210 nm to 290 nm at a 5 nm step size. The resulting wavelength-dependent profile of the Bzp derivatized PC 16 : 0/18 : 1 (9Z) showed two broad absorption bands at 210–225 nm and 255–270 nm of ^9^F_A_ ions (magenta trace in Figure 3(a)). However, only one broad band at 210–225 nm was observed for ^9^F_O_ ions (red trace in Figure 3(a)). The bands in the wavelength-dependent photodissociation profile with using 2-CF_3_-Bzp or 3-CF_3_-Bzp as the PB reagent were analogous to the main band in the profile of Bzp, besides some intensity variations of ^9^F_A_ ions from 255 nm to 270 nm (Figure S7). Interestingly, ^9^F_A_ ions were observed at high relative abundances for 4-CF_3_-Bzp over a wide range of 210–270 nm (magenta trace in Figure 3(b)). ^9^F_O_ ions were observed around 210–225 nm but at lower relative abundances in the wavelength-dependent photodissociation profile (red trace in Figure 3(b)). No C=C diagnostic fragment was observed when the wavelength is longer than 280 nm for all studied PB reagents. This suggested that the energy of a photon above 280 nm (≤4.4 eV) was not sufficient to initiate the dissociation of oxetane ring containing PB products to produce fragments.

In short, 4-CF_3_-Bzp was found as the best PB reagent for UVPD MS/MS, which had the following advantages: (1) the diagnostic ions were produced at relatively high abundances for unambiguous identification of C=C locations (Figures 2(f), 2(g), and 3(b)); (2) wavelength-dependent dissociation with UVPD MS/MS offers the opportunity to use a proper wavelength to initiate bond-selective dissociation, which produces characteristic diagnostic ions to easy C=C locations for shotgun lipidomics; (3) relatively high PB conversion was achieved compared with Bzp, 2-CF_3_-Bzp, and 3-CF_3_-Bzp (e.g., up to 40% for PC 16 : 0/18 : 1 (9Z) after only 12 s, Figure S8).

### 2.2. PB-UVPD MS/MS Analysis of PE, PG, PS, and PA

**Figure 4:**
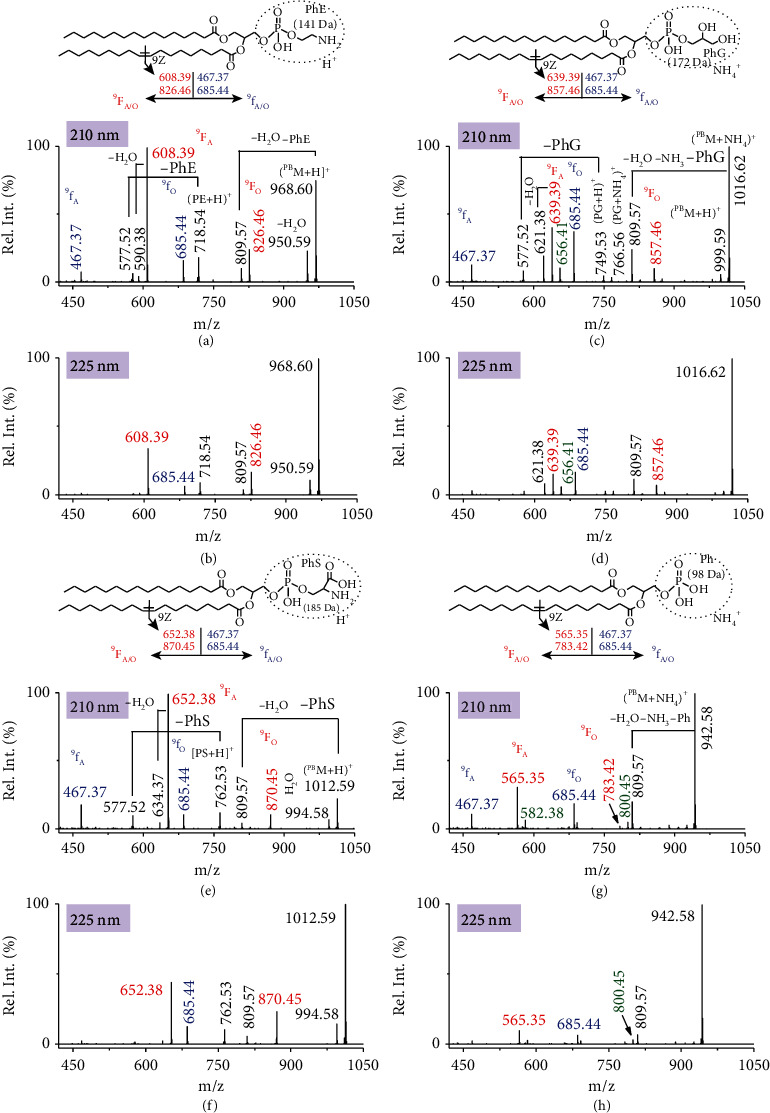
UVPD MS/MS spectra of the PB products of (a, b) PE 16 : 0/18 : 1 (9Z), (c, d) PG 16 : 0/18 : 1 (9Z), (e, f) PS 16 : 0/18 : 1 (9Z), or (g, h) PA 16 : 0/18 : 1 (9Z) with 4-CF_3_-Bzp at 210 nm and 225 nm after 2 pulses. Protonated diagnostic ion pair ^9^F_A/O_ of C=C bond was labelled as red, and the corresponding headgroup-liberated C=C diagnostic pair ^9^f_A/O_ was colored as blue. NH_4_^+^-cationized diagnostic ions were also observed for (c, d) PG and (g, h) PA and labelled as olive. The headgroups of PE, PG, PS, and PA were denoted as PhE, PhG, PhS, and Ph, respectively. Wavelength-dependent photodissociation profiles and pulse-dependent photodissociation efficiency of the PB products of PE 16 : 0/18 : 1 (9Z) with 4-CF_3_-Bzp can be found in Figure S9.

We further investigated the scope of coupling PB reaction using 4-CF_3_-Bzp with wavelength-tunable UVPD MS/MS to study different classes of GPLs. These included the synthetic standards of phosphatidylethanolamines (PEs), phosphatidylglycerols (PGs), phosphatidylserines (PSs), and phosphatidic acid (PAs) (Figure 4). When the 4-CF_3_-Bzp derivatized PE ions ([^PB^*M*+H]^+^, *m*/*z* 968.60) were subjected to 210 nm UVPD MS/MS analysis, two pairs of C=C diagnostic fragments were identified, each with a mass difference of 218 Da (Figure 4(a)). One pair of C=C diagnostic ions (^9^F_A/O_, *m*/*z* 608.39 and *m*/*z* 826.46) labelled as red in Figure 4(a) originated from the direct dissociation of oxetane rings on protonated PB products, and the other pair of fragments (^9^f_A/O_, *m*/*z* 467.37 and *m*/*z* 685.44) colored in blue derived from sequential loss of the headgroup (PhE) as a neutral. Besides, some neutral-loss peaks, such as the loss of −H_2_O (*m*/*z* 950.59), −H_2_O−PhE (*m*/*z* 809.57), –PB reagent (*m*/*z* 718.54), ^9^F_A_−H_2_O (*m*/*z* 590.38), or −PB reagent−PhE (*m*/*z* 577.52) were also observed. The 210 nm UVPD pattern of PE is analogous to the spectrum from CID MS/MS, but some neutral-loss fragments, such as −H_2_O and −H_2_O−PhE, were observed with higher relative ion abundances in the CID MS/MS spectrum (Figure S11). This comparison again indicated the bond-selective fragmentation of UVPD MS/MS. Surprisingly, when 225 nm UVPD was applied, the fragmentation was more selective toward the formation of the PhE containing C=C diagnostic fragments (^9^F_A/O_) and a suppression of neutral-loss peaks to be observed at a relative low intensity on the spectrum (Figure 4(b)). We hypothesized that the stereo- and regioisomers of the PB products resulting in formation of the neutral-loss products may have a lower photoabsorption cross section at ∼225 nm region. In addition, the ^9^f_A_ ions (*m*/*z* 467.37) due to a sequential neutral loss of PhE from ^9^F_A_ (*m*/*z* 608.39) was completely suppressed at 225 nm, whereas the corresponding ^9^f_O_ ions (*m*/*z* 685.44) were decreased as well, suggesting the ability to tune the degree of sequential fragmentation and simplify spectrum. The wavelength-dependent UVPD therefore would facilitate a higher sensitivity and confidence in the assignment of C=C locations.

NH_4_^+^-cationized PB products of PG 16 : 0/18 : 1 (9Z) with 4-CF_3_-Bzp reagent were subject to UVPD MS/MS analysis due to being of higher abundance than the protonated product ions. Two pairs of protonated C=C diagnostic fragments (^9^F_A/O_, *m*/*z* 639.39 and *m*/*z* 857.46 and ^9^f_A/O_, *m*/*z* 467.37 and *m*/*z* 685.44) were observed with high intensities by employing 210 nm UVPD MS/MS (Figure 4(c)). Additionally, NH_4_^+^ adducted ^9^F_A_ fragment (*m*/*z* 656.41) containing PhG headgroup was detected. A suppression of sequential fragmentation was also observed for the neutral-loss peaks at 225 nm UVPD MS/MS (Figure 4(d)). Like the corresponding PE 16 : 0/18 : 1 (9Z), PS 16 : 0/18 : 1 (9Z) was identified to give similar results in terms of wavelength-dependent fragmentation pattern (Figures 4(e) and 4(f)). For PA 16 : 0/18 : 1 (9Z), however, the 210 nm UVPD MS/MS produced prominent C=C diagnostic ion pairs, and almost no signal corresponding to neutral-loss peaks could be detected except for *m*/*z* 809.57 ions (Figure 4(g)). UVPD at 225 nm gave a similar fragmentation pattern to that of 210 nm UVPD accompanied by a reduced fragmentation efficiency (Figure 4(h)). The optimal wavelengths for PB-UVPD MS/MS analysis of GPLs with 4-CF_3_-Bzp reagent are tabulated in Table 1 along with the corresponding identified C=C diagnostic ions listed (Table 1). These results suggest that different lipid classes have different UVPD behaviour; thus, wavelength-tunable UVPD MS/MS would be desirable to be applied for sensitive identification of C=C positions in complex mixtures when coupling with PB reaction with 4-CF_3_-Bzp reagent.

### 2.3. Analysis of Unsaturated GPLs from Complex Lipid Extracts

**Figure 5:**
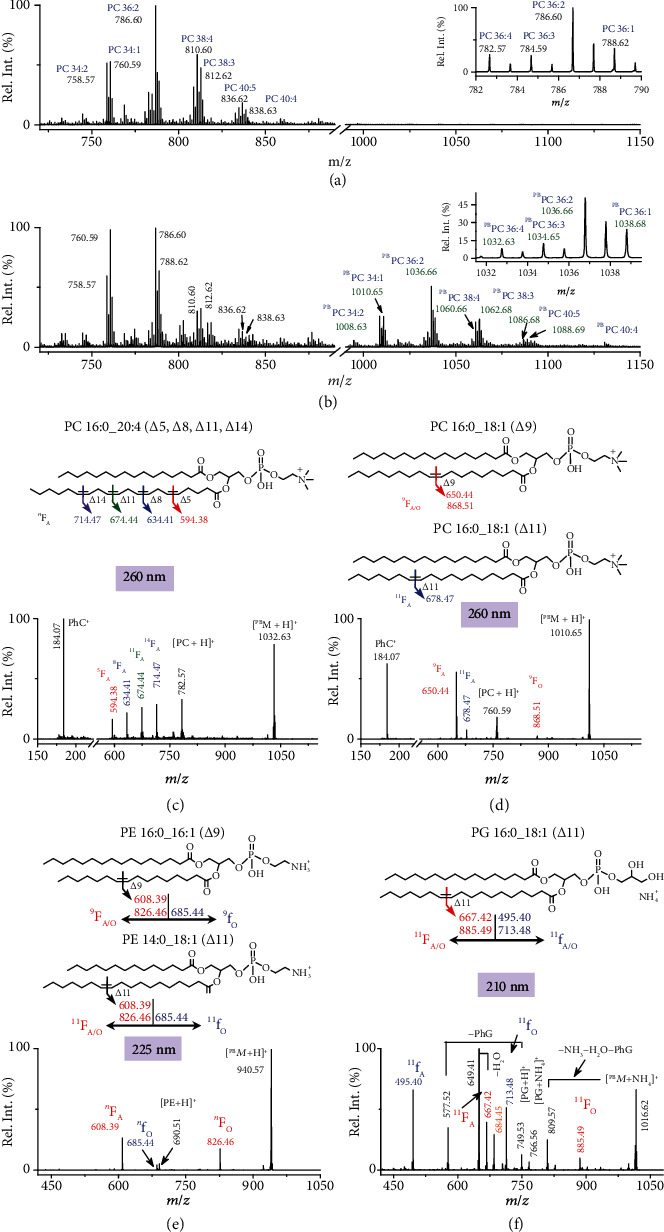
Profiles of unsaturated PCs and corresponding photoproducts of the bovine liver polar extract (a) before and (b) after PB reaction with 4-CF_3_-Bzp for 10 s. The PB products are annotated with “^PB^PC.” Zoomed-in spectra over the *m*/*z* range of 782–790 and 1031.5–1039.5 were shown in insets. All the labeled peaks were assigned as protonated PC lipids or photoproducts. UVPD MS/MS spectra of photoproducts of (c) PC 36 : 4 and (d) PC 34 : 1 at 260 nm and (e) PE 32 : 1 at 225 nm, as well as (f) PG 34 : 1 at 210 nm after 2 pulses. ∆*n* indicates the site(s) of unsaturation in the acyl chain, but the C=C geometry (viz cis/trans) is unknown.

The wavelength-tunable UVPD coupled with PB derivatization using 4-CF_3_-Bzp reagent was applied to commercially available polar lipid extracts from bovine liver using the shotgun approach. As shown in Figure 5(a), 10 unsaturated PC species, with carbon numbers in fatty acyls ranging from 34 to 40 and C=C numbers ranging from 1 to 5, were identified in the nano-ESI MS spectrum. The fatty acyl composition for each PC species was obtained from CID MS/MS of CH_3_COO^−^ adducted lipids in negative ion mode (Figure S12), leading to the identification of 13 groups of fatty acyl composition isomers of PCs (Table S1) [7]. For instance, PC 36 : 3 was identified as PC 16 : 0_20 : 3, PC 18 : 0_18 : 3, and PC 18 : 1_18 : 2, based on the clear detection of three pairs of fatty acyl anions (*m*/*z* 255.23 (C16:0) and 305.25 (C20:3), *m*/*z* 283.26 (C18:0) and 277.22 (C18:3), and *m*/*z* 281.25 (C18:1) and 279.23 (C18:2)) (Figure S12(d)). The *sn*-positions of fatty acyls could not be confidently determined.

The PB derivatization with 4-CF_3_-Bzp reagent led to 250 Da mass shift for unsaturated lipid, avoiding *m*/*z* overlap between derivatized PCs with intact ones of higher masses (Figure 5(b)). The resulting protonated photoproducts of PC species were mass-selected for UVPD MS/MS analysis. Besides the observation of PhC^+^ ions at *m*/*z* 184.07 to reconfirm the subclass of the extracted lipids, fragment ion *^n^*F_A_ characteristic to C=C locations was identified with photodissociation at 260 nm, in good accordance with the corresponding numbers of C=C bonds in the fatty acyls (Figure 5(c) and Figure S13(a–g)). For instance, the *^n^*F_A_ ions at *m*/*z* 594.38, *m*/*z* 634.41, *m*/*z* 674.44, and *m*/*z* 714.47 clearly suggested the existence of *Δ*5, *Δ*8, *Δ*11, and *Δ*14 C=C locations for the C20:4 chain of PC 16 : 0_20 : 4 (PC 36 : 4), thus identifying the lipid species as PC 16 : 0_20 : 4 (*Δ*5, *Δ*8, *Δ*11, and *Δ*14) (note that the corresponding *^n^*F_O_ ions could also be observed at low abundances when the region between *m*/*z* 800 and 975 has been magnified ×30, Figure S14). PB-UVPD MS/MS analysis of PC 16 : 0_18 : 1 from PC 34 : 1 ([^PB^*M*+H]^+^, *m*/*z* 1010.65) at 260 nm revealed that it consisted of two C=C location isomers as *Δ*9 and *Δ*11 in C18:1 based on the detection of two *^n^*F_A_ ions at *m*/*z* 650.44 and *m*/*z* 678.47, as well as ^9^F_O_ ions (*m*/*z* 868.51) at lower abundances (Figure 5(d)). According to the relative ion abundances of the observed ^9^F_A_ and ^11^F_A_ ions, PC 16 : 0_18 : 1 (*Δ*9) was estimated to be the major species among the lipid isomers. Similarly, PC 18 : 0_18 : 1 assigned from PC 36 : 1 was also determined to contain two C=C location isomers: PC 18 : 0_18 : 1 (*Δ*9) and PC 18 : 0_18 : 1 (*Δ*11) based on two *^n^*F_A_ ions at *m*/*z* 678.47 and *m*/*z* 706.50 (Figure S13(h)). The above identification could be further confirmed from 210 nm UVPD MS/MS (Figure S13). In total, 15 molecular species of unsaturated PCs in the bovine liver extracts were identified down to C=C locations (Table S1), comparable to that observed from 193 nm UVPD MS/MS on the Orbitrap mass spectrometer with high-resolution capabilities [24]. It is noteworthy that, in comparison to determine the fatty acyl compositions and C=C locations of PCs with separate steps, 193 nm UVPD MS/MS was found to be capable of characterizing fatty acyl compositions and C=C positions within one MS/MS spectrum of protonated lipids [24].

The above analysis strategy was also applied to lipid extracts from *E. coli* by direct infusion. Unsaturated PEs and PGs possessing 1–2 C=C bonds were identified. The resulting PB derivatized lipids modified with 4-CF_3_-Bzp were formed (Figure S15). The PE and PG compositions were determined via CID MS/MS of the deprotonated lipid anions (Figures S16 and S17) with subsequent UVPD MS/MS of the PB products generating diagnostic ions to locate the sites of C=C bonds. An example of the UVPD MS/MS results is illustrated in Figure 5(e) for the characterization of the C=C positions in PE 32 : 1. 225 nm UVPD MS/MS of the resulting PB products (*m*/*z* 940.57) generated a diagnostic ion pair *^n^*F_A/O_ to provide the C=C positions in PE 32 : 1. The UVPD spectrum also revealed the subclass of the studied lipid as PE based on mass difference of 141 Da of the observed fragment *^n^*f_O_ at *m*/*z* 685.44 relative to *^n^*F_O_ ions at *m*/*z* 826.46, which could be used as a signature to identify the headgroup. Combined with the compositions of the two constituent fatty acyl moieties in PE 32 : 1 as 16 : 0_16 : 1 and 14 : 0_18 : 1 provided with CID MS/MS (Figure S16(a)), PE 32 : 1 was allowed confident identification and localization of the C=C positions to be PE 16 : 0_16 : 1 (*Δ*9) and PE 14 : 0_18 : 1 (*Δ*11) (Figure 5(e) and Table S2). Similarly, detailed structural characterization in terms of the compositions of the two fatty acyl chains and the relevant double bond positions was also identified for PE 34 : 1, PE 34 : 2, PE 35 : 2, and PE 36 : 2 (Figures S16 and S18). It is noteworthy that only one pair of C=C diagnostic fragments was observed for two-double-bond-containing PE 34 : 2, PE 35 : 2, and PE 36 : 2, indicating the same position of the C=C bond in each fatty acyl counted from ester linkage, e.g., PE 16 : 1 (*Δ*9)_18 : 1 (*Δ*11). Moreover, in addition to PEs, PGs were also detected and identified by employing 210 nm UVPD MS/MS because of the high abundances of the photofragment ions associated with C=C bond locations (Figure 5(f), Figures S17 and S19). In the *E. coli* extract, 9 PE and 7 PG molecular species with C=C location assignment were identified (Table S2). Interestingly, the C=C sites in C16:1, C17:1, C18:1, and C19:1 in all PEs and PGs are exclusively at *Δ*9, *Δ*10, *Δ*11, and *Δ*12 positions, respectively, likely resulting from the process of GPL biosynthesis in *E. coli* whereby the enzyme FabA incorporates a unsaturation site at the 3-position of a 10-carbon intermediate prior to chain elongation [44]. This process leads to acyl chains with unsaturation elements consistently occurring at the same position (7 carbons) relative to the terminal methyl carbon.

## 3. Conclusions

In this study, a chromophore-tagging strategy combined with wavelength-tunable UVPD MS/MS has been explored in the identification of C=C location isomers of unsaturated lipids. PB reaction has been applied to incorporate chromophores to C=C moiety in fatty acyls, leading to the formation of photocleavable motifs with highly specific photoabsorption. This allows a degree of selectivity for fragmentation with UVPD MS/MS at wavelength longer than 200 nm. The wavelength-dependent photodissociation has been investigated in terms of generating abundant C=C diagnostic fragments. The combination of PB reaction with UVPD MS/MS appears powerful for C=C location isomer identifications of a variety of glycerophospholipid species from a complex lipid extract, such as bovine liver or *E. coli* extracts, using a shotgun approach. Additionally, selectivity and fragmentation efficiency of the developed strategy can be further improved by screening for PB reagents with larger absorption cross sections at the UVPD wavelength in use or varying the UVPD wavelength. The enhanced UVPD efficiency for generating C=C diagnostic fragments highlights the potential to effectively improve the identification of the C=C bond locations in unsaturated lipids and the potential to be implemented on a liquid chromatographic time scale.

## 4. Materials and Methods

### 4.1. Materials and Chemicals

All chemical reagents and solvents were purchased from commercial sources and used without further purification. Unsaturated phospholipids including PCs, PEs, PGs, PSs, and PAs were purchased from Avanti Polar Lipids, Inc. (Alabaster, AL, USA). The lipid polar extracts of bovine liver and *E. coli* were purchased from Avanti Polar Lipids (Alabama, U.S.A.). Bzp, Atp, and their −CF_3_-monosubstituted derivatives were obtained from Aladdin industrial. Inc. (Shanghai, China) and J&K Scientific Ltd. (Beijing). HPLC-grade acetonitrile and methanol were purchased from Thermo Fisher Scientific Inc. (Rockford, IL, USA).

### 4.2. Site-Specific Photochemical Reaction

Phospholipid standards were dissolved in acetonitrile/water (50/50, v/v) at 2–10 *μ*M and Bzp, Atp, or their −CF_3_-monosubstituted derivatives at 500 *μ*M. CH_3_COONH_4_ was also added at 10 mM to facilitate ionization in the subsequent step for MS analysis. The solution (200 *μ*L) was placed in a borosilicate glass vial (1.5 mL) and purged with N_2_ for 10 min to displace residual O_2_ dissolved in the solution before the photoreaction reaction. The solution was then pumped through a flow microreactor (in *μ*L/min flow rate range), made from UV-transparently coated fused silica capillary (5 cm, 100 *μ*m i.d., 375 *μ*m o.d., Polymicro Technologies, Phoenix, AZ, USA). A low-pressure mercury (LP-Hg) lamp with an emission band around 254 nm (BHK, Inc., Ontario, CA, USA) was used to initiate the photochemical reactions. The lamp was placed in parallel to the capillary at a distance of approximately 1 cm. At the exit of the microflow reactor, the reaction solution was collected in nanoESI tips for subsequent direct infusion MS for UVPD MS/MS analysis. The reaction time, also named UV exposure time, was adjusted between 2 s and 20 s by varying the flow rate. All photochemical reaction setups were covered with foil to prevent direct human exposure to UV light.

### 4.3. Mass Spectrometry and Ultraviolet Photodissociation

A Maxis Impact Q-TOF mass spectrometer (Bruker Daltonics, Bremen, Germany) has been modified to build ultraviolet photodissociation (UVPD, Figure S1). The quadrupole for collision, located between the quadrupole mass filter (QMF) and the pusher for orthogonal TOF, was modified to trap the ions selected by QMF. A wavelength-tunable laser beam, delivered by an OPO (optical parametric oscillator) laser source (NT342B, EKSPLA, Vilnius, Lithuania), was introduced through a CaF_2_ window installed the flange to the pusher region of the TOF analyzer. Then, the UV beam, e.g., 210 nm (5.9eV), 225 nm (5.5eV), or 260 nm (4.8eV), was guided to the linear ion trap (LIT) for fragmentation of the trapped ions with the help of a reflecting mirror (Thorlabs, Newton, NJ, United States) machined in the push plate assembly. The sequence applied to LIT was generated with a digital delay/pulse generator (Model DG645, Stanford Research Systems, CA, United States) independently without consideration of the delay of TOF analyzer. The other pulse generator (Model ZKG027, Kuancheng Electrical Engineering Co. Ltd., Hefei, China) was used to trigger the laser emission and DG645 to synchronise laser pulses with the trapping events. The pulse numbers of the laser into the LIT within a trapping event can be adjusted. The laser was allowed to operate at a maximum repetition rate of 20 Hz. The energies used in the ultraviolet range were 0.5–1.0 mJ per pulse. To obtain a wavelength-dependent photodissociation profile, mass-selected PB product ions were accumulated in the LIT for 50–300 ms and subjected to a laser at a given wavelength with corresponding 1–6 pulses for fragmentation after which the ions are scanned out of the LIT to record a product mass spectrum. Photoproduct yields are recorded over a range of wavelengths and collated to produce a photodissociation profile according to the wavelength. The resolutions of MS and UVPD MS/MS at low and high *m*/*z* have been given in Figure S20.

## Figures and Tables

**Table 1:** The wavelength used for PB-UVPD MS/MS analysis of GPLs with 4-CF_3_-Bzp reagent and the corresponding identified C=C diagnostic ions.

Lipid classes	UVPD wavelength	Diagnostic fragments for C=C locations
PC	210 nm	F_A_, F_O_
	260 nm	F_A_
PE	225 nm	F_A_, F_O_; f_A_
PG	210 nm	F_A_, F_O_; f_A_, f_O_
PS	225 nm	F_A_, F_O_; f_A_
PA	210 nm	F_A_, F_O_; f_A_, f_O_

## Data Availability

All data used to support the findings of this study are available from the corresponding author upon request.

## Supplementary Materials

Supplementary Materials Figure S1: schematic of the modified Q-TOF MS with wavelength-tunable UVPD capabilities. Figure S2: UVPD MS/MS of protonated intact PC 16 : 0/18 : 1 (9Z) at 260 nm after 10 pulses. Figure S3: relative intensities of parent and fragmentation ions with respect to pulse numbers of UVPD MS/MS of Paternò-Büchi photoproducts. (a) 210 nm and (b) 260 nm UVPD MS/MS of photoproduct [^PB^*M*+H]^+^ for the reaction of PC 16 : 0/18 : 1 (9Z) with Bzp; (c) 210 nm UVPD MS/MS of [^PB^*M*+H]^+^ for PC 16 : 0/18 : 1 (9Z) with 4-CF_3_-Bzp. Figure S4: UVPD MS/MS of product ions for the Paternò-Büchi reactions of PC 16 : 0/18 : 1 (9Z) with (a) 2-CF_3_-Bzp or (b) 3-CF_3_-Bzp at 210 nm and 260 nm after 2 pulses. Figure S5: UVPD MS/MS of product ions for the Paternò-Büchi reactions of methyl oleate with Bzp at (a) 210 nm and 260 nm (b) with 2 pulses. The time-dependent density functional theory calculations to predict UV-vis absorption spectra of Na^+^ adducted PB products for generating F_A_ and F_O_ ions are shown in panels (c) and (d). Figure S6: UVPD MS/MS of product ions for the Paternò-Büchi reactions of PC 16 : 0/18 : 1 (9Z) with (a) 2-CF_3_-Atp, (b) 3-CF_3_-Atp, or (c) 4-CF_3_-Atp at 210 nm and 260 nm after 2 pulses. Figure S7: wavelength-dependent relative ion intensities of parent and fragmentation ions for UVPD MS/MS of PB products of PC 16 : 0/18 : 1 (9Z) with (a) Bzp, (b) 2-CF_3_-Bzp, (c) 3-CF_3_-Bzp, and (d) 4-CF_3_-Bzp after 2 pulses. Figure S8: reaction-time dependent yield of Paternò-Büchi photoproducts of PC 16 : 0/18 : 1 (9Z) with (a) Bzp, (b) 2-CF_3_-Bzp, (c) 3-CF_3_-Bzp, and (d) 4-CF_3_-Bzp. Figure S9: (a) wavelength-dependent photodissociation profile of the PB products of PE 16 : 0/18 : 1 (9Z) with 4-CF_3_-Bzp after 2 pulses and pulse-dependent photodissociation efficiency at (b) 210 nm, (c) 225 nm, and (d) 260 nm. Figure S10: UVPD MS/MS spectrum of the PB product of PE 16 : 0/18 : 1 (9Z) with 4-CF_3_-Bzp at 220 nm after 2 pulses. Figure S11: CID MS/MS of (a) PE 16 : 0/18 : 1 (9Z), (b) PG 16 : 0/18 : 1 (9Z), (c) PS 16 : 0/18 : 1 (9Z), and (d) PA 16 : 0/18 : 1 (9Z) after Paternò-Büchi reaction with 4-CF_3_-Bzp. Figure S12: analysis of fatty acyl chains of 10 PCs from the bovine liver polar extract with CID MS/MS in negative mode. Figure S13: C=C bond locations of (a) PC 34 : 2, (b) PC 36 : 2, (c) PC 36 : 3, (d) PC 38 : 3, (e) PC 38 : 4, (f) PC 40 : 4, (g) PC 40 : 5, and (h) PC 36 : 1 from the bovine liver polar extract with 210 nm and 260 nm UVPD MS/MS after 2 pulses. Figure S14: 260 nm-UVPD MS/MS spectrum of PB product of PC 16 : 0_20 : 4 with 4-CF_3_-Bzp after 2 pulses. Figure S15: profiles of unsaturated PEs, PGs, and corresponding photoproducts of the *E. coli* lipid extracts (a) before and (b) after Paternò-Büchi reaction with 4-CF_3_-Bzp, respectively. Figure S16: analysis of fatty acyl chains of 5 PEs from the *E. coli* lipid extracts with CID MS/MS in negative mode. PEs labeled as red were assigned as [*M*−H]^−^. Figure S17: analysis of fatty acyl chains of 5 PGs from the *E. coli* lipid extracts with CID MS/MS in negative mode. Figure S18: C=C bond locations of (a) PE 34 : 2, (b) PE 34 : 1, (c) PE 35 : 2, and (d) PE 36 : 2 from the *E. coli* lipid extracts with 210 nm, 225 nm, and 260 nm UVPD MS/MS after 2 pulses. Figure S19: C=C bond locations of (a) PG 32 : 1, (b) PG 34 : 1, (c) PG 35 : 2, (d) PG 36 : 2, and (e) PG 37 : 2 from the *E. coli* lipid extracts with 210 nm, 225 nm, and 260 nm UVPD MS/MS after 2 pulses. Figure S20: resolutions of MS and UVPD MS/MS at low and high *m*/*z*. Table S1: C=C location isomers of PCs identified from bovine liver polar extract. Table S2: C=C location isomers of PEs and PGs identified from the *E. coli* lipid extracts.
