# Supplementary material for: Site-Specific Photochemical Reaction for Improved C=C Location Analysis of Unsaturated Lipids by Ultraviolet Photodissociation
Source: Research (Wash D C). 2022 Feb 12;2022:9783602. doi: 10.34133/2022/9783602 (PMC8859641; doi:10.34133/2022/9783602)
Supplement: Supplementary Materials — Figure S1: schematic of the modified Q-TOF MS with wavelength-tunable UVPD capabilities. Figure S2: UVPD MS/MS of protonated intact PC 16 : 0/18 : 1 (9Z) at 260 nm after 10 pulses. Figure S3: relative intensities of parent and fragmentation ions with respect to pulse numbers of UVPD MS/MS of Paternò-Büchi photoproducts. (a) 210 nm and (b) 260 nm UVPD MS/MS of photoproduct [PBM+H]+ for the reaction of PC 16 : 0/18 : 1 (9Z) with Bzp; (c) 210 nm UVPD MS/MS of [PBM+H]+ for PC 16 : 0/18 : 1 (9Z) with 4-CF3-Bzp. Figure S4: UVPD MS/MS of product ions for the Paternò-Büchi reactions of PC 16 : 0/18 : 1 (9Z) with (a) 2-CF3-Bzp or (b) 3-CF3-Bzp at 210 nm and 260 nm after 2 pulses. Figure S5: UVPD MS/MS of product ions for the Paternò-Büchi reactions of methyl oleate with Bzp at (a) 210 nm and 260 nm (b) with 2 pulses. The time-dependent density functional theory calculations to predict UV-vis absorption spectra of Na+ adducted PB products for generating FA and FO ions are shown in panels (c) and (d). Figure S6: UVPD MS/MS of product ions for the Paternò-Büchi reactions of PC 16 : 0/18 : 1 (9Z) with (a) 2-CF3-Atp, (b) 3-CF3-Atp, or (c) 4-CF3-Atp at 210 nm and 260 nm after 2 pulses. Figure S7: wavelength-dependent relative ion intensities of parent and fragmentation ions for UVPD MS/MS of PB products of PC 16 : 0/18 : 1 (9Z) with (a) Bzp, (b) 2-CF3-Bzp, (c) 3-CF3-Bzp, and (d) 4-CF3-Bzp after 2 pulses. Figure S8: reaction-time dependent yield of Paternò-Büchi photoproducts of PC 16 : 0/18 : 1 (9Z) with (a) Bzp, (b) 2-CF3-Bzp, (c) 3-CF3-Bzp, and (d) 4-CF3-Bzp. Figure S9: (a) wavelength-dependent photodissociation profile of the PB products of PE 16 : 0/18 : 1 (9Z) with 4-CF3-Bzp after 2 pulses and pulse-dependent photodissociation efficiency at (b) 210 nm, (c) 225 nm, and (d) 260 nm. Figure S10: UVPD MS/MS spectrum of the PB product of PE 16 : 0/18 : 1 (9Z) with 4-CF3-Bzp at 220 nm after 2 pulses. Figure S11: CID MS/MS of (a) PE 16 : 0/18 : 1 (9Z), (b) PG 16 : 0/18 : 1 (9Z), (c) [file 9783602.f1.zip › Li_Supporting_Information.docx]

**Supporting information for**

**Site-Specific Photochemical Reaction for Improved C=C Location Isomer Analysis of Unsaturated Lipids by Ultraviolet Photodissociation**

**Hai-Fang Li^1^, Jing Zhao^2^, Wenbo Cao^1^, Wenpeng Zhang^1^, Yu Xia^2^, and Zheng Ouyang^1^**

*^1^ State Key Laboratory of Precision Measurement Technology and Instruments, Department of Precision Instrument, Tsinghua University, Beijing 100084, China.*

*^2^ MOE Key Laboratory of Bioorganic Phosphorus Chemistry & Chemical Biology, Department of Chemistry, Tsinghua University, Beijing 100084, China.*

Correspondence should be addressed to Yu Xia; xiayu@tsinghua.edu.cn, and Zheng Ouyang; ouyang@tsinghua.edu.cn **Table of Contents**

Lipid Nomenclature S3

Quantum Chemistry Calculation. S3

Figure S1: Schematic of UVPD MS S4

Figure S2: UVPD MS/MS of intact PC 16:0/18:1 (9Z) S5

Figure S3: Pulse-number-dependent dissociation of PC photoproducts. S6

Figure S4: UVPD MS/MS of photoproduct of PC with -CF_3_-Bzp S8

Figure S5: Mechanism insight for F_A_ and F_O_ generation. S9

Figure S6: UVPD MS/MS of photoproduct of PC with Atp and -CF_3_-Atp S11

Figure S7: Wavelength-dependent photodissociation of PC with Bzp and -CF_3_-Bzp S12

Figure S8: Reaction-time dependent yield of Paternò-Büchi photoproducts S13

Figure S9: Wavelength-dependent photodissociation of PE with 4-CF_3_-Bzp and pulse-number-dependent dissociation efficiency S14

Figure S10: 220nm-UVPD MS/MS of PB products of PE with 4-CF_3_-Bzp S15

Figure S11: CID MS/MS of PB products of PE, PG, PS, and PA S16

Figure S12: Fatty acyl identification of PC with CID MS/MS in negative mode S17

Figure S13: C=C bond locations of PC with PB-UVPD MS/MS S19

Figure S14: 260nm-UVPD MS/MS of PB products of PC16:0_20:4 S19

Figure S15: Profiles of PEs, PGs and corresponding photoproducts of the *E. coli* lipid S20

Figure S16: Fatty acyl identification of PEs with CID MS/MS in negative mode S21

Figure S17: Fatty acyl identification of PGs with CID MS/MS in negative mode S22

Figure S18: C=C bond locations of PEs with PB-UVPD MS/MS S23

Figure S19: C=C bond locations of PGs with PB-UVPD MS/MS S24

Figure S20: Resolutions of MS and UVPD MS/MS S25

Table S1: C=C Location Isomers of PCs Identified from Bovine Liver Polar Extract S26

Table S2:C=C Location Isomers of PEs and PGs Identified from the *E. coli* lipid extracts S28

References S29

**Lipid Nomenclature**

Shorthand notations for structural annotations of lipids are adopted from LIPID MAPS [1, 2]. For example, PC 16:0/18:1 (9Z) indicates a phosphatidylcholine (PC) lipid containing two fatty acyl chains with 16 carbons and 18 carbons on *sn*-1 and *sn*-2 position, respectively. The “0” and “1” after the carbon number refers to the degree of unsaturation of each fatty acyl chain. The position of C=C in the fatty acyl chain is defined in parentheses. “9Z” is used to indicate the site(s) of unsaturation in the acyl chain, where “9” corresponds to the ninth of carbon atom counted from ester linkage and “Z” indicates the C=C geometry as cis. When “∆*n*” is labled, meaning that the C=C geometry (viz cis/trans) is unknown. A forward slash “/” is used to separate the known *sn*-1 and *sn*-2 fatty acyl chains with the *sn*-1 chain placed before “/” while underscore “_” suggests that the *sn*-position of fatty acyl chains is unspecified.

Quantum Chemistry Calculation.

Density functional theory (DFT) calculations were carried out using the Gaussian 09 program package [3] for geometry optimizations of PB product isomers. The M06-2X functional [4] was adopted with 6-31++G(d,p) basis sets [5] for Na, C, O, and H atoms. The final optimized geometries were characterized as the minima by performing vibrational frequency calculations to confirm that each of the geometries has zero imaginary frequency. The zero-point vibration corrected energies (Δ*H*_0_) and the free energies at 298 K (Δ*G*_298_) relative to the separated reactants [FAME 18:1(∆9) + Na^+^ + Bzp] were reported in unit of kcal∙mol^−1^. The time-dependent DFT (TD-DFT) calculations [6] were performed on the DFT optimized geometries of PB product isomers with the B3LYP functional [7, 8] to predict UV-vis absorption spectra.

Figure S1: Schematic of the modified Q-TOF MS with wavelength-tunable UVPD capabilities

Figure S2: UVPD MS/MS of protonated intact PC 16:0/18:1 (9Z) at 260 nm after 10 pulses

Figure S3: Relative intensities of parent and fragmentation ions with respect to pulse numbers of UVPD MS/MS of Paternò-Büchi photoproducts. (a) 210 nm and (b) 260 nm UVPD MS/MS of photoproduct [^PB^*M* + H]^+^ for the reaction of PC 16:0/18:1 (9Z) with Bzp; (c) 210 nm UVPD MS/MS of [^PB^*M* + H]^+^ for PC 16:0/18:1 (9Z) with 4-CF_3_-Bzp.

In order to obtain diagnostic ions F_A_ and F_O_ with high abundances for C=C locations, pulse-dependent photodissociation of PB products was conducted for UVPD MS/MS of photoproduct [^PB^*M* + H]^+^ of PC 16:0/18:1 (9Z) with Bzp, or 4-CF_3_-Bzp. From the pulse-dependent measurements (Figures S3(a) and 3(b)), the relative intensities of photoproduct [^PB^*M* + H]^+^ decreased significantly along with the increase of pulse numbers applied for 210 nm- and 260 nm-UVPD MS/MS of [^PB^*M* + H]^+^. The resulting PhC^+^ fragment, corresponding to phosphocholine ions, increased significantly. However, when the pulse number was increased to be 2, the high relative intensities of diagnostic ion F_A_ and F_O_ have been observed. Minor increases were observed when further increasing the pulse numbers from 2 to 4 for 210 nm-UVPD MS/MS or 2 to 10 for 260nm-UVPD MS/MS of PB products of PC 16:0/18:1 (9Z) with Bzp. When the pulse number was increased from 4 to 10 for 210 nm-UVPD MS/MS of Bzp derivatized PC 16:0/18:1 (9Z), the relative ion intensities of F_A_ ions became decreased gradually, indicating the generated F_A_ ions begin to undergo secondary fragmentation. Like the reagent of Bzp, 4-CF_3_-Bzp was identified to give a similar result in terms of pulse-dependent profile of relative ion intensities of parent and fragments for 210 nm-UVPD MS/MS of PB products of PC 16:0/18:1 (9Z) with 4-CF_3_-Bzp (Figures S3(c)).

Figure S4: UVPD MS/MS of product ions for the Paternò-Büchi reactions of PC 16:0/18:1 (9Z) with (a) 2-CF_3_-Bzp, or (b) 3-CF_3_-Bzp at 210 nm (left panels) and 260 nm (right panels) after 2 pulses. C=C bond diagnostic ions denoted as F_A_ and F_O_ were labeled as olive and orange, respectively. Phosphocholine ions at *m/z* 184.08 were denoted as PhC^+^. The signal marked by asterisks in Fig S4(b) (left panel) was due to the contribution of O_2_ and other impurity in the reaction system.

Figure S5: UVPD MS/MS of product ions for the Paternò-Büchi products of methyl oleate (FAME 18:1 (Δ9)) with Bzp at (a) 210 nm and 260 nm (b) after 2 pulses. The time-dependent density functional theory (TD-DFT) calculations to predict UV-vis absorption spectra of Na^+^ adducted PB products for generating F_A_ and F_O_ ions are shown in panels (c) and (d). Insets to the panels (c) and (d) show the lowest-lying energy isomers of PB product isomers for generating F_A_ and F_O_ ions, respectively. Δ*H*_0_ (Δ*G*_298_) relative to the separated reactants [FAME 18:1(∆9) + Na^+^ + Bzp] were also shown below in each structure.

When Bzp or 4-CF_3_-Bzp was used as a reagent, similar results were observed for the relative ion intensities of *I*(F_A_)/*I*(F_O_) at 210 nm and 260 nm-UVPD MS/MS. The relative intensity of *I*(F_A_)/*I*(F_O_) is higher at 260 nm than that at 210 nm. To support the assumptions that the difference in fragment ion intensity ratio of F_A_/F_O_ are due to difference in molar UV absorptivity of PB product isomers, methyl oleate (FAME 18:1 (Δ9)) was used as a model compound to examine UV absorptivity of its PB product isomers without time-consuming to determine the lowest-lying energy isomers of PB products. UVPD MS/MS of benzophenone derivatized FAME 18:1 (Δ9) gave the similar results associated with the tendency of the relative ion intensities of F_A_ and F_O_ ions at 210 nm and 260 nm (Figures S5(a) and S5(b)). The *I*(^9^F_A_)/*I*(^9^F_O_) was around 16.0 from 210 nm-UVPD MS/MS while it went up to about 171 under 260 nm. Two types of PB products, P1 and P2, are formed due to different orientations of the PB reagent relative to the lipid C=C bond upon [2+2] cycloaddition (Figure 1 in the main text) for generating F_A_ and F_O_ diagnostic ions from UVPD MS/MS. Density functional theory (DFT) calculations were conducted to study the structures of PB product isomers (see Theoretical Methods in the Supporting Information). The structures IS1 and IS2 with Na^+^-ion-bonded two O atoms in oxetane moiety and carbonyl linkage through noncovalent interactions were proposed as the lowest-lying energy isomers for generating F_A_ and F_O_ ions, respectively (insets in Figures S5(c) and S5(d)). The adopted M06-L functional predicts that the energy of IS1 structure (∆*H*_0_: −51.4 kcal∙mol^−1^) which is the precursor of F_A_ ions is lower than that of IS2 isomer (∆*H*_0_: −44.4 kcal∙mol^−1^), the precursor of F_O_ ions, suggesting that the population of IS1 isomer is more than that of IS2 in the ion trap for UVPD. Moreover, time-dependent DFT (TD-DFT) calculations on the IS1 and IS2 geometries predicted that the absorption intensities of the IS1 and IS2 isomers at 210 nm is 0.498 and 0.601, respectively, indicating that the dissociation efficiency of IS2 isomer to produce F_O_ ions at 210 nm-UVPD MS/MS maybe higher than that of IS1 isomer to produce F_A_ ions. However, when considering the population of IS1 and IS2 isomers in the ion trap, the relative intensities of F_A_ ions from IS1 is still higher than that of F_O_ ions from IS2 (*I*(F_A_)/*I*(F_O_)=16.0). When the applied wavelength for UVPD MS/MS moved from 210 nm to 260 nm, the absorption intensities of IS1 isomer at 260 nm was calculated to be higher than that of IS2 isomer (0.342 vs 0.265). So the relative intensities of F_A_ ions from IS1 would be much higher than that of F_O_ ions from IS2. *I*(F_A_)/*I*(F_O_) was experimentally characterized to be 171 at 260 nm-UVPD, higher than that of *I*(F_A_)/*I*(F_O_) at 210 nm-UVPD (*I*(F_A_)/*I*(F_O_)=16.0), which is consistent with the theoretical calculations. These results can be used to explain and extend the experimental observation gained from the study of PC 16:0/18:1 (9Z) with Bzp or 4-CF_3_-Bzp.

Figure S6: UVPD MS/MS of product ions for the Paternò-Büchi reactions of PC 16:0/18:1 (9Z) with (a) 2-CF_3_-Atp, (b) 3-CF_3_-Atp, or (c) 4-CF_3_-Atp at 210 nm (left panels) and 260 nm (right panels) after 2 pulses. C=C bond diagnostic ions denoted as F_A_ and F_O_ were labeled as olive and orange, respectively. Phosphocholine ions at *m/z* 184.08 were denoted as PhC^+^.

Figure S7: Wavelength-dependent relative ion intensities of parent and fragmentation ions for UVPD MS/MS of PB products of PC 16:0/18:1 (9Z) with (a) Bzp, (b) 2-CF_3_-Bzp, (c) 3-CF_3_-Bzp, and (d) 4-CF_3_-Bzp after 2 pulses.

Figure S8: Reaction-time dependent yields of Paternò-Büchi photoproducts of PC 16:0/18:1 (9Z) with a) Bzp, b) 2-CF_3_-Bzp, c) 3-CF_3_-Bzp, and d) 4-CF_3_-Bzp. Yield = *I* ([^PB^*M* + H]^+^) / [*I* ([^PB^*M* + H]^+^) + *I* ([PC + H]^+^)]

Figure S9: (a) Wavelength-dependent photodissociation profile of the PB products of PE 16:0/18:1 (9Z) with 4-CF_3_-Bzp after 2 pulses and pulse-dependent photodissociation efficiency at (b) 210 nm, (c) 225 nm, and (d) 260 nm. Total F_A_: *I* (*m/z* 608.39 + *m/z* 590.38 + *m/z* 467.37); Total F_O_: *I* (*m/z* 826.46 + *m/z* 685.44); Total [PE + H]^+^: *I* (*m/z* 718.54 + *m/z* 577.52); Total [^PB^*M* + H]^+^: *I* (*m/z* 968.60 + *m/z* 950.59 + *m/z* 809.57). The assignments of *m/z* have been shown in Figure 4 in the main text.

In Figure S9(a), a total of F_A_ ions at 220 nm were observed to be at a highest intensity from UVPD MS/MS of PB products of PE 16:0/18:1 (9Z) with 4-CF_3_-Bzp. However, in the 220nm-UVPD MS/MS spectra shown in Figure S10, two pairs of C=C diagnostic fragments were observed. One pair of C=C diagnostic ions (^9^F_A/O_) labelled as red originated from the direct dissociation of oxetane rings on protonated PB products, and the other pair of fragments (^9^f_A/O_) colored in blue derived from sequential loss of the headgroup (PhE) as a neutral. Besides, some neutral loss peaks were also observed. When 225 nm-UVPD was applied, the fragmentation was more selective toward the formation of the PhE containing C=C diagnostic fragments (^9^F_A/O_) and a suppression of neutral-loss peaks to be observed at a relative low intensity on the spectrum. 225 nm-UVPD MS/MS had the capability for C=C location characterization of PEs, which has been confirmed by lipid analysis from complex lipid extracts. Considering that 225 nm-UVPD MS/MS spectra permits C=C location of PEs for easy interpretation and unambiguous identification without existing database searching tools, the wavelength of 225 nm was recommended for analyzing C=C locations of PEs.

Figure S10: UVPD MS/MS spectrum of the PB product of PE 16:0/18:1 (9Z) with 4-CF_3_-Bzp at 220 nm after 2 pulses. Two pairs of C=C diagnostic ions assigned ^9^F_A/O_ and ^9^f_A/O_ were observed.

Figure S11: CID MS/MS of (a) PE 16:0/18:1 (9Z), (b) PG 16:0/18:1 (9Z), (c) PS 16:0/18:1 (9Z), and (d) PA 16:0/18:1 (9Z) after Paternò-Büchi reaction with 4-CF_3_-Bzp. C=C bond diagnostic ions were labeled as red, blue or olive. Collision energies (CEs) applied for PE, PS, and PA were 25 eV, and CE for PG was 10 eV. Neutral losses of H_2_O from PB products were highlighted in yellow in each panels.

Figure S12: Analysis of fatty acyl chains of 10 PCs from the bovine liver polar extract with CID MS/MS in negative mode. PCs assigned as [*M* + CH_3_COO]^−^ were labeled as red and diagnostic ion pairs of fatty acyls were labeled as olive, blue, or purple.

Figure S13: C=C bond locations of (a) PC 34:2, (b) PC 36:2, (c) PC 36:3, (d) PC 38:3, (e) PC 38:4, (f) PC 40:4, (g) PC 40:5, (h) PC 36:1 from the bovine liver polar extract with 210 nm (top panels) and 260 nm (below panels) UVPD MS/MS after 2 pulses.

Figure S14: 260 nm-UVPD MS/MS spectrum of PB product of PC16:0_20:4 with 4-CF_3_-Bzp after 2 pulses. The range of *m/z* 800−975 was magnified ×30.

Figure S15: Profiles of unsaturated PEs, PGs and corresponding photoproducts of the *E. coli* lipid extracts (a) before and (b) after Paternò-Büchi reaction with 4-CF_3_-Bzp, respectively. The Paternò-Büchi products are annotated with “^PB^PE” and “^PB^PG”. The spectrum in panel b has been enlarged 2 times over the *m/z* range of 900−1100. The labeled PEs and PGs were assigned as [*M* + H]^+^ and [*M* + NH_4_]^+^, respectively. The reaction time was 10 s.

Figure S16: Analysis of fatty acyl chains of 5 PEs from the *E. coli* lipid extracts with CID MS/MS in negative mode. PEs labeled as red were assigned as [*M* − H]^−^. Diagnostic ion pairs of fatty acyls were labeled as olive and blue.

Figure S17: Analysis of fatty acyl chains of 5 PGs from the *E. coli* lipid extracts with CID MS/MS in negative mode. PGs labeled as red were assigned as [*M* − H]^−^. Diagnostic ion pairs of fatty acyls were labeled as olive and blue.

Figure S18: C=C bond locations of (a) PE 34:2, (b) PE 34:1, (c) PE 35:2, and (d) PE 36:2 from the *E. coli* lipid extracts with 210 nm (top panels), 225 nm (middle panels) and 260 nm (below panels) UVPD MS/MS after 2 pulses.

Figure S19: C=C bond locations of (a) PG 32:1, (b) PG 34:1, (c) PG 35:2, (d) PG 36:2, and (e) PG 37:2 from the *E. coli* lipid extracts with 210 nm (top panels), 225 nm (middle panels) and 260 nm (below panels) UVPD MS/MS after 2 pulses. The diagnostic ions F_A_ labeled as purple were assigned as NH_4_^+^-cationized fragments, and diagnostic ion pairs labeled as red and blue were assigned as protonated fragments.

Figure S20: Resolutions of MS and UVPD MS/MS at low and high *m/z*

Table S1: C=C Location Isomers of PCs Identified from Bovine Liver Polar Extract. Mass accuracies for the identified ions have been given in parentheses.

| Identified PCs | [M + CH_3_COO]^−^ (*m/z*) | Fatty acyl ions  (*m/z*) | [^PB^*M* + H]^+^  (*m/z*) | C=C diagnostic ions  (*m/z*) |
| --- | --- | --- | --- | --- |
| PC 16:0_18:2 (∆9, ∆12) | 816.58 (12 ppm) | 255.23 (21 ppm), 279.23 (20 ppm) | 1008.63 (10 ppm) | 650.44 (16 ppm), 868.51 (14 ppm);  690.47 (18 ppm), 908.54 (17 ppm) |
| PC 16:0_18:1 (∆9) | 818.59 (9 ppm) | 255.23 (19 ppm), 281.25 (18 ppm) | 1010.65  (8 ppm) | 650.44 (18 ppm), 868.51 (15 ppm) |
| PC 16:0_18:1 (∆11) | 818.59 (9 ppm) | 255.23 (19 ppm), 281.25 (18 ppm) | 1010.65  (8 ppm) | 678.47 (17 ppm), 896.54 (16 ppm) |
| PC 16:0_20:4  (∆5, ∆8, ∆11, ∆14) | 840.58 (11 ppm) | 255.23 (25 ppm), 303.23 (21 ppm) | 1032.63  (7 ppm) | 594.38 (19 ppm), 812.45 (15 ppm); 634.41 (18 ppm), 852.48 (14 ppm); 674.44 (17 ppm), 892.51 (14 ppm);  714.47 (17 ppm), 932.54 (13 ppm) |
| PC 16:0_20:3  (∆8, ∆11, ∆14) | 842.59 (13 ppm) | 255.23 (22 ppm), 305.25 (20 ppm) | 1034.65  (9 ppm) | 636.42 (18 ppm), 854.50 (13 ppm); 676.46 (17 ppm), 894.53 (13 ppm); 716.49 (17 ppm), 934.56 (12 ppm) |
| PC 18:0_18:3  (∆6, 9, 12) | 842.59 (13 ppm) | 283.26 (21 ppm), 277.22 (23 ppm) | 1034.65  (9 ppm) | 636.42 (18 ppm), 854.50 (13 ppm); 676.46 (17 ppm), 894.53 (13 ppm); 716.49 (17 ppm), 934.56 (12 ppm) |
| PC 18:1 (∆12)_18:2 (∆9, ∆12) | 842.59 (13 ppm) | 281.25 (21 ppm), 279.23 (20 ppm) | 1034.65  (9 ppm) | 676.46 (17 ppm), 894.53 (13 ppm);  716.49 (17 ppm), 934.56 (12 ppm) |
| PC 18:0_18:2 (∆9, 12) | 844.61 (15 ppm) | 283.26 (17 ppm), 279.23 (17 ppm) | 1036.66 (10 ppm) | 678.47 (15 ppm), 896.54 (13 ppm);  718.50 (14 ppm), 936.57 (13 ppm) |
| PC 18:0_18:1 (∆9) | 846.62 (10 ppm) | 283.26 (20 ppm), 281.25 (21 ppm) | 1038.68  (8 ppm) | 678.47 (18 ppm), 896.54 (15 ppm) |
| PC 18:0_18:1 (∆11) | 846.62 (10 ppm) | 283.26 (20 ppm), 281.25 (21 ppm) | 1038.68  (8 ppm) | 706.50 (16 ppm), 924.57 (13 ppm) |
| PC 18:0_20:4  (∆5, ∆8, ∆11, ∆14) | 868.61 (8 ppm) | 283.26 (23 ppm), 303.23 (21 ppm) | 1060.66  (6 ppm) | 622.41 (15 ppm), 840.48 (12 ppm); 662.44 (15 ppm), 880.51 (12 ppm); 702.47 (14 ppm), 920.54 (10 ppm); 742.50 (13 ppm), 960.57 (9 ppm) |
| PC 16:0_22:4  (∆7, ∆10, ∆13, ∆16) | 868.61 (8 ppm) | 255.23 (21 ppm), 331.26 (19 ppm) | 1060.66  (6 ppm) | 622.41 (15 ppm), 840.48 (12 ppm); 662.44 (15 ppm), 880.51 (12 ppm); 702.47 (14 ppm), 920.54 (10 ppm); 742.50 (13 ppm), 960.57 (9 ppm) |
| PC 18:0_20:3  (∆8, ∆11, ∆14) | 870.62 (11 ppm) | 283.26 (18 ppm), 305.25 (17 ppm) | 1062.68  (8 ppm) | 664.46 (18 ppm), 882.53 (16 ppm); 704.49 (17 ppm), 922.56 (15 ppm); 744.52 (17 ppm), 962.59 (12 ppm) |
| PC 18:0_22:5  (∆7, ∆10, ∆13, ∆16, ∆19) | 894.62 (7 ppm) | 283.26 (20 ppm), 329.25 (20 ppm) | 1086.68  (6 ppm) | 650.44 (15 ppm), 868.51 (12 ppm); 690.47 (15 ppm), 908.54 (12 ppm); 730.50 (13 ppm), 948.57 (13 ppm); 770.53 (13 ppm), 988.60 (10 ppm); 810.57 (14 ppm), 1028.64 (11 ppm) |
| PC 18:0_22:4  (∆7, ∆10, ∆13, ∆16) | 896.64 (9 ppm) | 283.26 (19 ppm), 331.26 (24 ppm) | 1088.69 (10 ppm) | 650.44 (16 ppm), 868.51 (18 ppm); 690.47 (16 ppm), 908.54 (18 ppm); 730.50 (14 ppm), 948.57 (15 ppm); 770.53 (14 ppm), 988.60 (16 ppm) |

Table S2: C=C Location Isomers of PEs and PGs Identified from the *E. coli* lipid extracts. Mass accuracies for the identified ions have been given in parentheses.

| Identified PEs | [M − H]^−^  (*m/z*) | Fatty acyl ions  (*m/z*) | [^PB^*M* + H]^+^  (*m/z*) | C=C diagnostic ions  (*m/z*) |
| --- | --- | --- | --- | --- |
| PE 16:0_16:1 (∆9) | 688.49 (10 ppm) | 253.23 (19 ppm), 255.21 (17 ppm) | 940.57 (8 ppm) | 608.39 (13 ppm),  826.46 (10 ppm) |
| PE 14:0_18:1 (∆11) | 688.49 (10 ppm) | 227.10 (16 ppm), 281.25 (21 ppm) | 940.57 (6 ppm) | 608.39 (13 ppm),  826.46 (10 ppm) |
| PE 16:1 (∆9)_18:1 (∆11) | 714.51 (12 ppm) | 253.21 (18 ppm), 281.25 (16 ppm) | 966.58 (9 ppm) | 634.41 (16 ppm),  852.48 (13 ppm) |
| PE 17:1 (∆10)_17:1 (∆10) | 714.51 (12 ppm) | 267.23 (18 ppm), 267.23 (18 ppm) | 966.58 (10 ppm) | 634.41 (16 ppm),  852.48 (13 ppm) |
| PE 16:0 _18:1 (∆11) | 716.52 (8 ppm) | 255.23 (18 ppm), 281.25 (22 ppm) | 968.60 (11 ppm) | 636.42 (15 ppm),  854.50 (12 ppm) |
| PE 17:1 (∆10)_18:1 (∆11) | 728.52 (12 ppm) | 267.23 (23 ppm), 281.25 (21 ppm) | 980.60 (7 ppm) | 648.42 (15 ppm),  866.50 (13 ppm) |
| PE 16:1 (∆9)_19:1 (∆12) | 728.52 (12 ppm) | 253.21 (18 ppm), 295.26 (24 ppm) | 980.60 (7 ppm) | 648.42 (15 ppm),  866.50 (13 ppm) |
| PE 18:1 (∆11)_18:1 (∆11) | 742.54 (15 ppm) | 281.25 (16 ppm), 281.25 (16 ppm) | 994.62 (8 ppm) | 662.44 (15 ppm),  880.51 (11 ppm) |
| PE 17:1 (∆10)_19:1 (∆12) | 742.54 (15 ppm) | 267.23 (18 ppm), 295.26 (20 ppm) | 994.62 (8 ppm) | 662.44 (15 ppm),  880.51 (11 ppm) |
| Identified PGs | [M − H]^−^  (*m/z*) | Fatty acyl ions  (*m/z*) | [^PB^*M* + NH_4_]^+^  (*m/z*) | C=C diagnostic ions  (*m/z*) |
| PG 16:0_16:1 (∆9) | 719.49 (12 ppm) | 255.21 (21 ppm), 253.23 (20 ppm) | 988.59 (9 ppm) | 639.39 (19 ppm),  857.46 (16 ppm) |
| PG 14:0_18:1 (∆11) | 719.49 (12 ppm) | 227.10 (21 ppm), 281.25 (19 ppm) | 988.59 (9 ppm) | 639.39 (19 ppm),  857.46 (16 ppm) |
| PG 16:0_18:1 (∆11) | 747.52 (9 ppm) | 255.23 (22 ppm), 281.25 (17 ppm) | 1016.62 (6 ppm) | 667.42 (17 ppm),  885.49 (15 ppm) |
| PG 17:1 (∆10)_18:1 (∆11) | 759.52 (17 ppm) | 267.43 (19 ppm), 281.25 (18 ppm) | 1028.62 (10 ppm) | 679.42 (17 ppm),  897.49 (14 ppm) |
| PG 16:1 (∆9)_19:1 (∆12) | 759.52 (17 ppm) | 253.21 (19 ppm), 295.26 (15 ppm) | 1028.62 (10 ppm) | 679.42 (17 ppm),  897.49 (14 ppm) |
| PG 18:1 (∆11)_18:1 (∆11) | 773.53 (14 ppm) | 281.25 (18 ppm), 281.25 (18 ppm) | 1042.64 (7 ppm) | 693.43 (19 ppm),  911.51 (16 ppm) |
| PG 18:1 (∆11)_19:1 (∆12) | 787.55 (15 ppm) | 281.25 (17 ppm), 295.26 (15 ppm) | 1056.65 (7 ppm) | 707.45 (18 ppm),  925.52 (13 ppm) |

**References**

[1] LIPID MAPS® Lipidomics Gateway. <http://www.lipidmaps.org>.

[2] G. Liebisch, J. A. Vizcaino, H. Koefeler et al., "Shorthand notation for lipid structures derived from mass spectrometry," *Journal of Lipid Research*, vol. 54, no. 6, pp. 1523−1530, 2013. DOI: 10.1194/jlr.M033506.

[3] M. J. Frisch, G. W. Trucks, H. B. Schlegel et al., "Gaussian 09, Revision A.1", Gaussian Inc., Wallingford, CT. 2009.

[4] Y. Zhao, and D. G. Truhlar, "The M06 suite of density functionals for main group thermochemistry, thermochemical kinetics, noncovalent interactions, excited states, and transition elements: two new functionals and systematic testing of four M06-class functionals and 12 other functionals." *Theoretical Chemistry Accounts*, vol. 120, no. 1, pp. 215–241, 2008. DOI: 10.1007/s00214-007-0310-x.

[5] V. A. Rassolov, M. A. Ratner, J. A. Pople, P. C. Redfern, and L. A. Curtiss, "6-31G* basis set for third-row atoms." *Journal of Computational Chemistry*, vol. 22, no. 9, pp. 976–984, 2001. DOI: 10.1002/jcc.1058.

[6] C. Adamo, and D. Jacquemin, "The calculations of excited-state properties with time-dependent density functional theory." *Chemical Society Reviews*, vol. 42, no. 3, pp. 845–856, 2013. DOI: 10.1039/C2CS35394F.

[7] A. D. Becke, "Density-functional thermochemistry. III. the role of exact exchange." *The Journal of Chemical Physics*, vol. 98, no. 7, pp. 5648–5652, 1993. DOI: 10.1063/1.464913.

[8] C. Lee, W. Yang, and R. G. Parr, "Development of the colle-salvetti correlation-energy formula into a functional of the electron density." *Physical Review B*, vol. 37, no. 2, pp. 785–789, 1988. DOI: 10.1103/PhysRevB.37.785.
